# Diagnostic accuracy of ultrasound for localising peripherally inserted central catheter tips in infants in the neonatal intensive care unit: a systematic review and meta-analysis

**DOI:** 10.1007/s00247-022-05379-7

**Published:** 2022-05-05

**Authors:** Shauna C. Doyle, Niamh M. Bergin, Rena Young, Andrew England, Mark F. McEntee

**Affiliations:** Discipline of Medical Imaging & Radiation Therapy, ASSERT Building, Brookfield Health and Sciences Complex, School of Medicine, University College of Cork, Cork, T12 AK24, Ireland

**Keywords:** Catheterization, Infant, Intensive care unit, Neonate, Peripherally inserted central catheter, Radiography, Ultrasound

## Abstract

**Background:**

Chest radiography after peripherally inserted central catheter insertion in infants is the reference standard method for verifying catheter tip position. The utilisation of ultrasound (US) for catheter placement confirmation in the neonatal and paediatric population has been the focus of many recent studies.

**Objective:**

In this systematic review we investigated the diagnostic accuracy of US for peripherally inserted central catheter tip confirmation in infants in the neonatal intensive care unit (NICU)

**Materials and methods:**

We conducted a systematic literature search of multiple databases. The study selection yielded eight articles, all of which had acceptable quality and homogeneity for inclusion in the meta-analysis. Sensitivity and specificity values were reported together with their respective 95% confidence intervals (CI).

**Results:**

After synthesising the eligible studies, we found that US had a sensitivity of 95.2% (95% CI 91.9–97.4%) and specificity of 71.4% (95% CI 59.4–81.6%) for confirming catheter tip position.

**Conclusion:**

Analyses indicated that US is an excellent imaging test for localising catheter tip position in the NICU when compared to radiography. Ultrasonography is a sensitive, specific and timely imaging modality for confirming PICC tip position. In cases where US is unable to locate malpositioned PICC tips, a chest or combined chest–abdominal radiograph should be performed.

## Introduction

Peripherally inserted central catheters (PICC) are ubiquitous in the neonatal intensive care unit (NICU) and are used to facilitate administration of total parenteral nutrition in low-birth-weight infants, and long-term administration of intravenous antibiotics [1]. The establishment of long-term venous access is also advantageous because it averts the need for repetitive catheterisation, thereby reducing pain and risk of infection for the infant whilst limiting excessive stimuli and extended time away from dedicated nursing care [2].

The use of PICCs in clinical practice is associated with various complications. Most of these complications are associated with malpositioning or migration of the catheter tip [3–5]. If the catheter tip is incorrectly placed, or migrates following insertion, pericardial effusion, thrombosis, infection, perforation, arrhythmia, cardiac tamponade, heart valve damage and patient discomfort can occur [6, 7].

Accurate PICC tip positioning is essential to minimise the risk of complications. The international standard for upper body inserted PICC tip position is at the caval–atrial junction at the lower third of the superior vena cava [8]. For PICCs inserted via the lower limb, the tip should be within the upper inferior vena cava between the 9th and 11th thoracic vertebrae [9, 10]. PICC tip localisation and confirmation can be achieved through myriad diagnostic imaging modalities. Verification of the catheter tip in infants is typically undertaken using the reference standard for PICC confirmation, an anteroposterior chest radiograph for upper-body-deployed PICCs and a combined chest–abdominal radiograph for PICCs deployed via the lower limb [11, 12].

There is evidence to suggest that chest radiography alone is not entirely accurate at identifying intra-atrial tip position [13, 14]. Additionally, it is often difficult to position the neonate for an optimum radiographic image without geometric or movement artefact. Chest radiography also provides only a static image of PICC position. Arm movements have been shown to influence the position of the PICC, so a radiograph captures the tip position in respect to the limb position of the neonate at the time of exposure and might not be entirely representative of the long-term position [15, 16].

Repositioning of misplaced PICC lines requires supplementary radiographs to re-assess tip position. This exposes the infant to additional ionising radiation repeatedly, until the catheter tip is in the appropriate position. Neonatal and paediatric populations are considerably more susceptible to ionising radiation because the rate at which their cells undergo mitosis is more rapid than that observed in adult populations [17]. Increased radiosensitivity, greater mitotic activity and a protracted period for consequences to manifest mean that the risk of radiation-induced cancer per unit of dose is 2–3 times higher for preterm infants than the average population [18]. Reduction in the risk of radiation-associated comorbidities can be achieved by limiting unnecessary radiographs by employing alternative methods to achieve similar diagnostic information or conclusions [19–21].

Bedside radiographic imaging in the NICU can be challenging for radiographers because the environment can make it impractical to comply with standard examination protocols. Neonates are unable to control their movements, making positioning accuracy difficult to accomplish. It is often the case that various lines, tubes and devices overlie the area of interest, interfering with image quality and diagnosis [22].

Neonatal intensive care unit staff are often required to be within 2 m of the X-ray source to hold the neonate, with their hands sometimes inadvertently being caught within the primary X-ray beam. A study by Russell et al. [23] showed that 15–40% of mobile radiographic examinations conducted in the NICU had at least one adult finger visible on the resultant image. In addition to direct radiation exposure, staff and patients in the NICU may also be exposed to secondary scatter radiation. Employing alternative imaging modalities that avoid the use of ionising radiation, such as US, is becoming increasingly popular, particularly for routine checks of catheter or tube positioning [24–26].

Technological advancements have expanded the role that US can play in the critical care setting through improved image quality and accessibility. Advantages of US include real-time assessment and limited handling of critically ill infants. Evidence shows that minimal training is required to reliably perform quantitative US [27]. Additionally, clinical reporting can be performed by the sonographer at the time of scanning, which can reduce report turnaround time. However, US does carry a higher infection control risk and therefore good basic hygiene standards are essential [28].

Previous systematic reviews [29, 30] have shown the success of US in localising central venous catheters in comparison to the reference standard (radiography), but these studies have not investigated the accuracy of US in a neonatal population. The aim of this systematic review was to identify, critically appraise and assess the sensitivity and specificity of US in confirming PICC tip position in infants on the NICU.

## Materials and methods

Using the Cochrane protocol, we conducted and report a systematic review (CRD42020223684) in adherence with the preferred reporting items for systematic reviews and meta-analyses (PRISMA) criteria/guidelines [31].

### Search strategy

We searched Embase, PubMed, CINAHL and MEDLINE (Ovid) in February 2021 and included eligible publications between January 2001 and February 2021. We also performed a grey literature search using OpenGrey and Google Scholar. We examined the bibliographies and reference lists of the identified studies for relevance and to identify additional studies. Two authors (S.C.D., N.M.B.), who were final year master’s-level student radiographers, independently searched the databases by applying a set of pre-determined search terms as described in Table 1.

**Table 1:** Summary of key search terms using population, interventions, comparators and outcomes (PICO)

PICO criteria	Search terms
Population	(NEONAT* *or* NEWBORN* *or* INFANT* *or* PREM*) *and*
	(“PERIPHERALLY INSERTED CENTRAL CATHETER” *or* PICC) *and*
Intervention	(ULTRAS* *or* ECHO* *or* SONOG* *or* “POINT OF CARE ULTRASOUND” *or* POCUS) *and*
Comparator	Radiography (NB—*not used as search term*)
Outcome	(LOCA* *or* PLACEMENT* *or* CONFIRM*)

### Study selection

Two authors (S.C.D., N.M.B.) independently screened titles and abstracts of the returned search results to determine applicability for inclusion. Articles were included if they were published in English during the last 10 years and assessed the diagnostic accuracy of US for PICC tip confirmation in infants in the NICU. References of the chosen studies were exported to a bibliographic database through EndNote [32] and duplicates were automatically removed. All studies eligible for inclusion or classified as “unclear” based on the title or abstract were subjected to a full-text review by either author. Full texts were independently assessed for eligibility using the pre-set inclusion and exclusion criteria. Discrepancies between the two authors were resolved by consensus discussion.

### Risk of bias assessment

We assessed the internal validity of the included studies using the Quality Assessment of Diagnostic Accuracy Studies-2 tool (QUADAS-2) [33]. The quality assessment of homogeneity was completed by both authors, independently. The tool assesses bias across four separate domains: patient selection, index test, reference test, and flow and timing. Concerns regarding the applicability of the study were judged dependent on whether risk of bias was low, high or unclear across these four domains.

### Data abstraction

Two authors (S.C.D., N.M.B.) independently extracted data from articles meeting criteria for final inclusion. Any disagreement between the authors was mediated through consensus discussion. The designed data extraction tool collected elements relating to various aspects of the studies such as patient demographics, index test characteristics, methods and outcomes, study characteristics and diagnostic accuracy measurements. Extraction also included an assessment of the role and type of US within each study, for example where US was used to provide real-time intra-procedural guidance by the operator or whether US was used to evaluate a previously implanted PICC. Where available, we also recorded US technique, e.g., transthoracic, transoesophageal. We used Microsoft Excel (Microsoft Inc., Redmond, WA) for data management.

### Data analysis

The meta-analysis was conducted using RevMan 5 [34]. The diagnostic accuracy measures used in the analyses were sensitivity, specificity and positive and negative predictive values. A 2 × 2 contingency table was derived to represent the pooled diagnostic accuracy measurements across all included studies. Sensitivity and specificity figures of individual studies were summarised and compared using a forest plot. Sensitivity and specificity values are presented together with their corresponding 95% confidence intervals (CI). The diagnostic accuracy of US in assessing PICC tip location is summarised using the receiver operating characteristic curve (ROC). We constructed an ROC for each study as well as an ROC to represent the pooled diagnostic accuracy data.

## Results

**Fig. 1:**
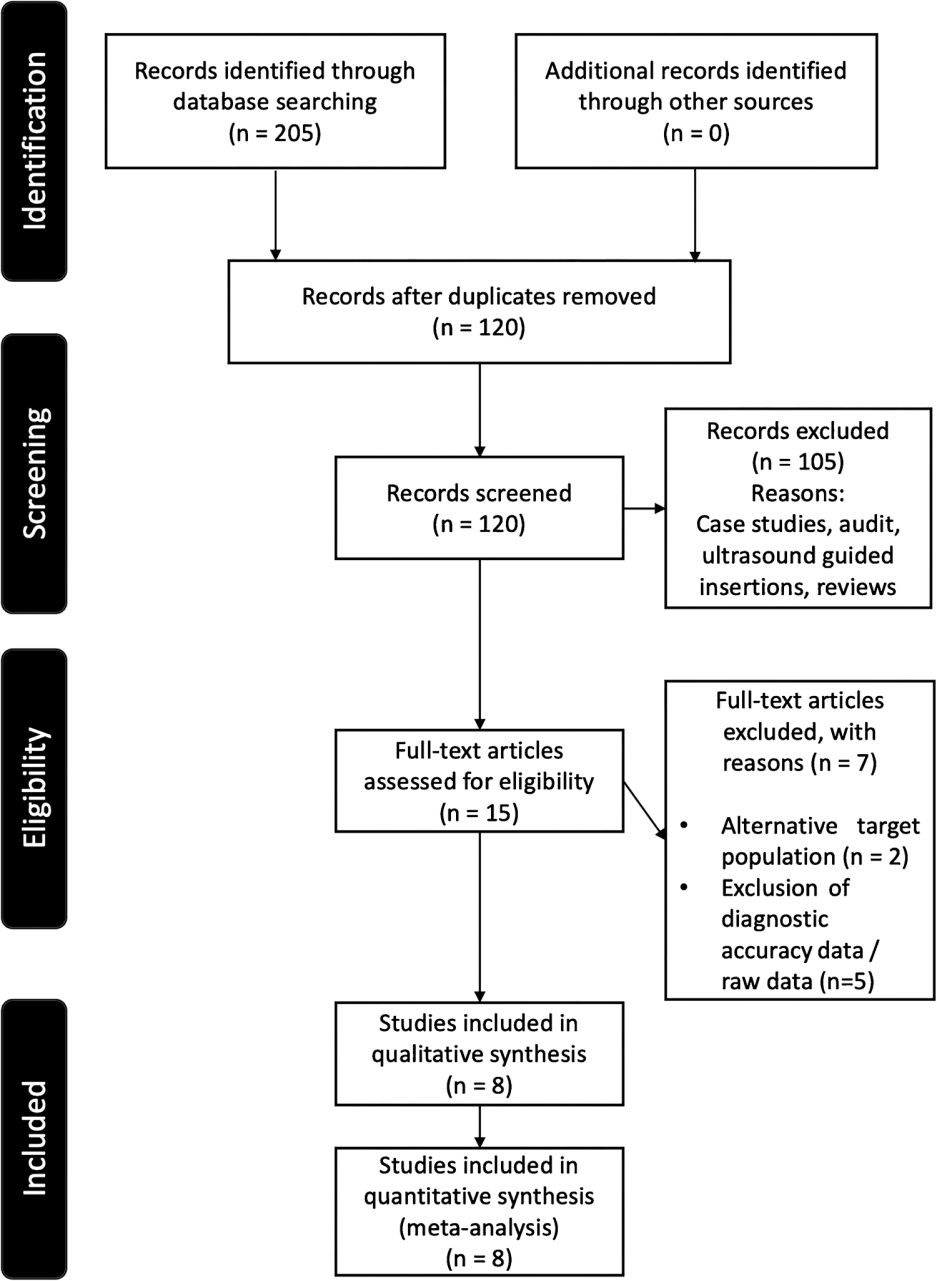
Preferred reporting items for systematic reviews and meta-analyses (PRISMA) flowchart summarises the review process and included studies

The PRISMA flowchart (Fig. 1) summarises the results of the search and review process. The search strategy yielded 120 studies following the removal of duplicates. We screened the abstracts and titles of these 120 papers and completed a full-text review of 15 studies. Eight papers were deemed eligible for inclusion. Potential studies for inclusion in this systematic review were appraised using the modified QUADAS-2 tool [33] (Table 2; [5, 35–41]). All eight studies were included because they had an overall low risk of bias.

**Table 2:** Analysis (risk of bias) outcome of the eight included studies using the modified QUADAS-2 tool [33]

Author(s)	Year	*n*	Patient selection	Index test: ultrasound	Reference standard	Flow and timing
Jain et al. [39]	2012	22	Low	Low	Low	Low
Kadivar et al. [36]	2020	90	Low	Low	Low	Low
Motz et al. [38]	2019	30	High	Low	Low	Low
Motz et al. [41]	2019	14	High	Low	Low	Low
Ren et al. [5]	2021	186	Low	Low	High	High
Saul et al. [35]	2016	25	Low	Low	Low	Unclear
Shabeer et al. [40]	2021	71	Low	Low	Low	Low
Telang et al. [37]	2017	31	Low	Unclear	Low	Low

*QUADAS* quality assessment of comparative diagnostic accuracy studies

Details of the study characteristics for all eight papers are detailed in Table 3 [5, 35–41]. Across the eight studies, 421 infants received a PICC line and 341 US scans were conducted. All eight studies reported the gestation for those neonates included, with a mean gestation for the eight studies of 38.6 (range 25.4 to 35.0) weeks. The mean birth weight recorded for included neonates was 1,581.1 (range 525.0 to 3,302.0) grams. All infants included in the study were in-patients in the NICU.

**Table 3:** Data extraction — US type and operator experience

Author(s)	US technique	Operator experience
Jain et al. [39]	Targeted echocardiography (UL, subcostal, apical, long parasternal views; LL, abdominal parasagittal view). 10-MHz multi-frequency probe	2 sonographers, extensive clinical research/training in targeted neonatal echocardiography
Kadivar et al. [36]	Post-insertion bedside US. 3–18 MHz linear phased-array and 1–10 MHz phased-array probes. No specific technique details stated	Radiologist with support from a paediatric cardiologist
Motz et al. [38]	UL – 13–6 MHz and 8–4 MHz linear phased-array probes. B-mode US protocol (transverse subclavicular long-axis, parasternal sagittal long-axis right and subcostal long-axis view. LL – 8–4 MHz phased-array probe. Right-side flank and standard subcostal echocardiography views	Neonatal–perinatal fellow who had performed > 50 US examinations prior to and 100 US during the study
Motz et al. [41]	13–6 MHz linear phased-array B-mode US protocol (transverse subclavicular long-axis, parasternal sagittal long-axis right ventricular inflow and subcostal long-axis view. 8–4 MHz for subcostal views	Diagnostic radiologist performed upper extremity PICC insertions. Paediatric cardiologist performed US examinations for PICCs in the lower SVC or heart
Ren et al. [5]	Post-insertion US. Multifrequency 6–15 MHz US probe with inferior vena cava and superior vena cava views	Not reported
Saul et al. [35]	12–5 MHz linear probe. Anatomical survey with transverse, sagittal and cine images of trachea, thymus, lungs and pleura, great vessels, aortic arch, heart. Dedicated transverse and sagittal views of the tip and shaft of the PICC	Neonatology resident with 4 years’ experience in US or attending radiologist with subspecialisation in sonography (~ 30 years)
Shabeer et al. [40]	13-MHz linear probe. Right parasternal axis and subcostal views	2 neonatology residents with 18 months of neonatal training and who perform regular US examinations of the brain and heart
Telang et al. [37]	5–12 MHz sector probe. Vena caval views and additional views for confirmation	Not reported

*LL* lower limb, *PICC* peripherally inserted central catheter, *SVC* superior vena cava, *UL* upper limb, *US* ultrasound

Five studies detailed the PICC insertion site, with 215 (59.9%) in the upper limb and 103 (28.7%) in the lower limb; 38 (10.6%) were superficial temporally located and three (0.8%) were auricle. Three studies (*n* = 64) did not report the exact insertion site of the PICC lines, but each of the three studies reported inclusion of both upper- and lower-extremity PICC line insertions [3, 21, 35].

All included studies reported diagnostic accuracy of US in relation to the reference standard. All included studies used radiography as the reference standard. The reference standard in six studies was solely chest radiography. In two studies the reference standard was both supine chest and abdominal radiography [36, 37].

Six studies provided details regarding the qualification and level of experience of the US operator involved (Table 3). The frequencies, brand and type of the US probes and machines used in the eight studies are reported in Table 4 [5, 35–41].

**Table 4:** Data extraction — index test characteristics

Author(s)	US equipment	Probe frequency	Probe type	Time	Blinded operator US	Success location of PICC (%)
Jain et al. [39]	Vivid I (GE Healthcare)	10 MHz	Multi-frequency	Not reported	Yes	100
Kadivar et al. [36]	Kontron Medical, Imagic Agile	3–18 MHz 1–10 MHZ	Linear or phased array	Not reported	Yes	81.1
Motz et al. [38]	S-ICU (Sonosite)	UE: 6–13 MHz LE: 4–8 MHz	UE: Linear & phased LE: Phased	LE: 5–10 min UE: 10–15 min	Yes	100
Motz et al. [41]	S-ICU (Sonosite)	UE: 6–13 MHz LE: 4–8 MHz	UE: Linear & phased LE: Phased	< 5 min SVC/BCV 5–10 min SVC/RA	Yes	100
Ren et al. [5]	Voluson (GE Healthcare)	6–15 MHz	Not reported	Not reported	Yes	100
Saul et al. [35]	iU22 (Philips Healthcare)	5–12 MHz 5–8 MHz^a^ 5–17 Hz^a^	Linear Curved^a^	Mean 7 min	Yes	91.0
Shabeer et al. [40]	Vivid 6 (GE Healthcare)	13 MHz	Linear	60 (35–108) min	Yes	95.0
Telang et al. [37]	Esaote	5–12 MHz	Sector	Not reported	Unclear	93.9

*BCV* brachiocephalic vein, *LE* lower extremity, *Min* minutes, *PICC* peripherally inserted central catheter, *RA* right atrium, *SVC* superior vena cava, *UE* upper extremity, *US* ultrasound

^a^ Supplementary images

All included studies acquired the reference standard image using a mobile radiography machine, although they did not specify the machine manufacture or detector type. All studies had a radiologist report the radiographs, except one, in which a neonatologist reported the radiographic images [37].

Four studies documented that the reference test was reported incorrectly. These erroneous radiographic reports were ultimately corrected following the US examination, which demonstrated a more accurate tip position [36, 38–40]. Radiographic misinterpretations in these four studies were a mixture of false reports of malposition and false reports of optimum position. Diagnostic accuracy measurements from included studies are summarised in Table 5 [5, 35–41].

**Table 5:** Diagnostic accuracy measurements for the eight included studies

Author(s)	Sensitivity	Specificity	PPV	NPV	TP	FP	TN	FN
Jain et al. [39]	60 (41.6–81.1)	58 (34.1–73.5)	55 (27.7–84.8)	64 (26.2–87.8)	6	5	7	4
Kadivar et al. [36]	100	89.5 (68.4–97.1)	97.3 (90.5–99.8)	100 (80.5–100)	71	0	17	2
Motz et al. [38]	97 (91.0–99.3)	66.0 (22.3–96.7)	98.0 (93.6–99.3)	57.0 (27.7–82.3)	91	2	4	3
Motz et al. [41]	100 (88.4–100)	100 (29.2–100.0)	100	100	11	0	3	0
Ren et al. [5]	100 (59.0–100)	100 (15.8–100)	100	100	30	0	2	0
Saul et al. [35]	47.8 (35.8–60.1)	100 (29.2–100)	100	100	7	0	3	0
Shabeer et al. [40]	47.8 (35.8–60.1)	82.4 (61.7–93.1)	78.6 (49.2–95.3)	53.8 (33.4–73.4)	14	12	11	3
Telang et al. [37]	96.6 (82.2–99.4)	100 (30.5–100.0)	100 (89.5–100)	75 (20.3–95.9)	28	1	3	1

*FN* false negative, *FP* false positive, *NPV* negative predictive value, *PPV* positive predictive value, *TN* true negative, *TP* true positive

**Fig. 2:**
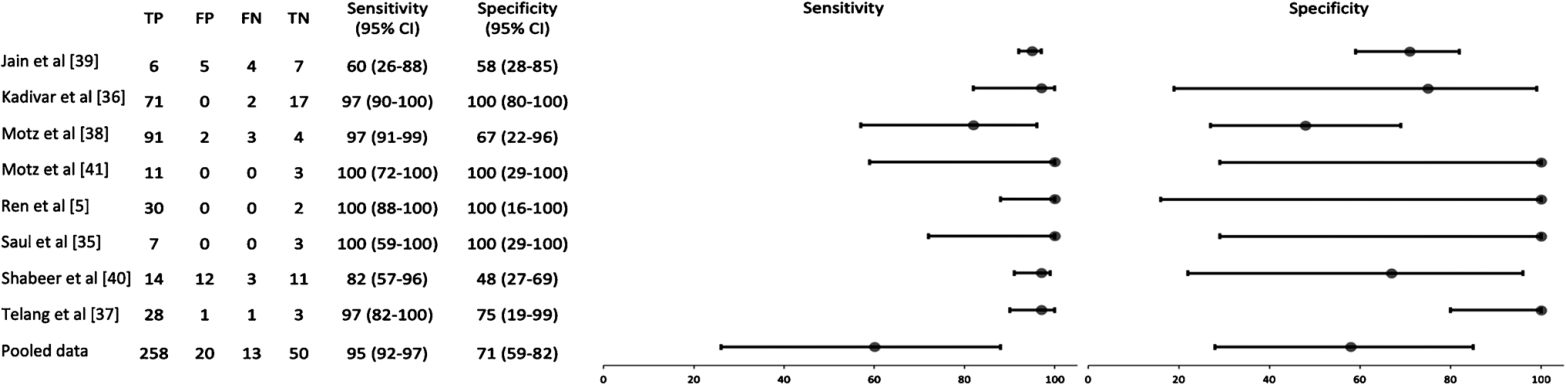
Forest plot presents data from the eight included studies. The ninth study represents the current study. The dots represent the estimated point for the odds ratio, and the line represents the 95% confidence interval

**Fig. 3:**
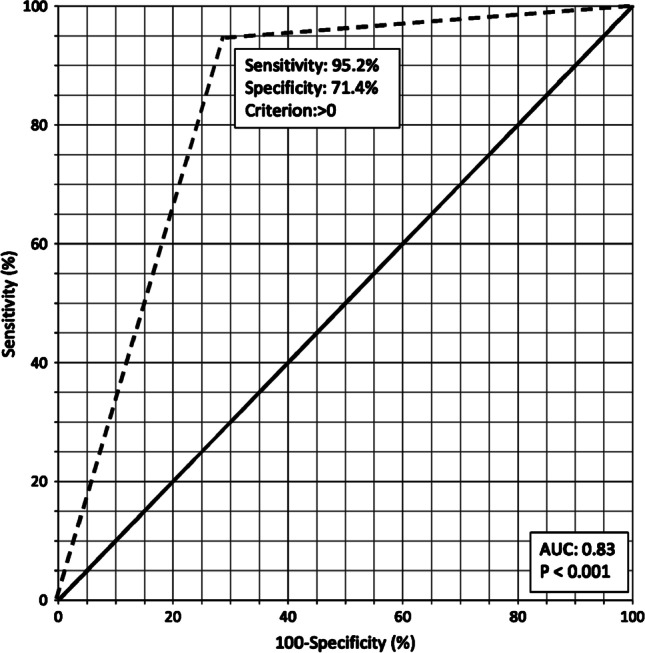
Receiver operating curve for pooled diagnostic accuracy indices. Area under the curve (AUC) = 0.83 (95% confidence interval: 0.79–0.87). The solid black line indicates a theoretical diagnostic test with a random performance level. The dotted black line indicates the performance of ultrasound in the confirmation of peripherally inserted central catheter placement

The estimated sensitivity and specificity for US were 95.2% (95% CI: 91.9–97.4%) and 71.4% (CI: 59.4–81.6%), respectively. The pooled positive likelihood ratio was 3.3 (95% CI: 2.3–4.8) and the negative likelihood ratio was 0.07 (95% CI: 0.04–0.12). The positive predictive value and the negative predictive value were 92.8% (95% CI: 89.9–94.9) and 6.7% (0.04–12.0), respectively. Figure 2 shows the forest plot of the overall odds ratios. The ROC curve for the pooled diagnostic accuracy of US across all eight studies indicated an acceptable overall accuracy (area under the curve [AUC] = 0.83) (Fig. 3).

## Discussion

Mobile radiographic imaging is the modality employed in the NICU for confirming PICC tip location. An imaging pathway free from ionising radiation, such as US, is a contemporary and novel approach to confirming the position of the PICC line tip in a neonatal population. By acknowledging the limitations of conventional radiography, it is reasonable to suggest that questions might soon be raised regarding the preferred primary imaging approach. This systematic review considers whether US is a viable alternative to mobile radiography in the localisation of the PICC line tip in a neonatal population.

Eight studies were eligible for inclusion in this review and meta-analysis. We calculated a pooled sensitivity of 95.2% (95% CI: 91.9–97.4%). The specificity values across all eight studies were variable because of misinterpretations made by the reference standard, lowering the pooled specificity value to 71.4% (95% CI: 59.4–81.6%). These results suggest US to be an excellent test for the localisation of the correctly positioned PICC line tip; however, it is less than perfect for identifying a misplaced or migrated PICC tip.

The low specificity can be explained by referencing the studies by Jain et al. [39] and Shabeer et al. [40]. Jain et al. [39] reported sensitivity and specificity values of 60% (95% CI 41.6–81.1) and 58% (95% 34.1–73.5%), respectively. Radiography inaccurately interpreted 4/11 misplaced PICC tips as appropriately placed, evidently impacting the false-negative rate, which had a negative impact on the sensitivity value. Furthermore, radiography incorrectly indicated that five of the correctly positioned PICC line tips were malpositioned, subsequently elevating the false-positive rate and negatively affecting the specificity. The study by Shabeer et al. [40] had a notably lower sensitivity value of 47.8% (95% CI 35.8–60.1%) in comparison to the other studies included in the analysis. The specificity value reported by Shabeer et al. was similar to that reported in the other studies (82.4% [95% CI 61.7–93.1%]). The limited specificity of US in detecting the PICC line tips in the study by Shabeer et al. [40] was also attributed to an imperfect reference standard. Chan et al. [42] acknowledged the variability amongst radiologists regarding the cavo-atrial junction, which would explain the misjudgements made on the reports in the studies by Jain et al. [39] and Shabeer et al. [40]. Taking the limitations of the reference standard into consideration, had both these studies been omitted from the meta-analysis, the pooled sensitivity and specificity values for the remaining seven studies would have been 97.5% and 91.4%, respectively.

The pervasiveness of PICC tip malposition within our systematic review aligns with that reported in the published literature in respect to the general population (10–53%) [43, 44]. Repositioning of these malpositioned or migrated catheter tips can be achieved under US guidance, avoiding the need for repeated radiographic imaging to reassess catheter tip location. This advantage of US has the potential to negate additional radiation exposure to the neonate [2].

Whilst our systematic review investigates the pooled diagnostic accuracy measurements of US as a confirmatory method for PICC line tip position in the neonatal population, our results align with recent publications exploring the accuracy of US for various other catheter and tube types. In 2017, a meta-analysis by Ablordeppey et al. [30] reported a pooled sensitivity of 82% (77–86%) and specificity of 98% (97–99%) of US as a confirmatory tool for central venous catheters. Similarly, a systematic review and meta-analysis published by Smit et al. [29] showed US to be an excellent alternative for the localisation of misplaced or migrated central venous catheter tips, reporting a pooled sensitivity and specificity of 68.2% (54.4–79.4%) and 98.9% (97.8–99.5%), respectively. The low sensitivity value was explained by an incorrect reference standard, further encouraging investigation into the accuracy of the reference standard chest radiography.

Our review extracted data in relation to the time taken to confirm PICC location using US. Four of the eight studies did not provide any data for the time taken to achieve PICC confirmation using US; however, all four of these studies reported that the US was faster than the reference standard. The remaining four studies reported time values. Three reported a scan time of less than 15 min [35, 39, 41]. The fourth study recorded a mean scan time of 60 min, which was considerably less than the time taken for conventional radiography (136 min) [40]. This element of the data extraction confirmed that US is a faster imaging modality than radiography for confirming catheter tip position in the neonatal population. In addition to this, US provides a real-time assessment, negating the time traditionally required for radiology reporting of the radiographic images.

Ultrasound quality is highly operator-dependent, making it necessary that US providers are appropriately trained. The lack of implementation of US as a contemporary imaging modality within the NICU setting is often a result of the lack of availability of appropriately qualified US staff [45]. The level of training of US operators varied across all eight studies included in our review. Six of the eight studies reported the qualification and training of the US operators. Despite varying levels of operator experience across all eight studies, diagnostic accuracy measurements did not vary significantly.

### Limitations

The quality of US examinations is highly operator-dependent and sonographic scans can be subject to interpretation bias. Errors in US examinations can occur during the study, in the form of bias and at the level of the individual operator through misinterpretation of clinical evidence, resulting in an erroneous diagnosis and affecting the clinical management of the individual patient. Six studies included in the review reported the qualifications and training received by the US practitioner. However, two studies did not report this information, which might have impacted on diagnostic accuracy.

Within recent years, US technology has been making considerable advances in the areas of image quality, image acquisition and physics [46]. Application of a language restriction to include only studies published in the English language might have limited our review. Considering the advancements in US science it is possible that additional research was published following the completion of our literature search. However, we are not aware of any new research published since execution of our data search and it is unlikely that a single additional publication would significantly impact our findings.

It is likely that the US visualisation of catheter tips and relevant anatomy varies depending on the deployment site. Anecdotally, it is likely to be easier to obtain a longitudinal view of the inferior vena cava and the inferior cavo-atrial junction with clear determination of the catheter tip when compared to obtaining longitudinal views of the superior vena cava for thoracic cage and pulmonary artefacts. It is therefore possible that if this review contained more lower-body PICCs, the sensitivity and specificity rates might be artificially higher. Sufficient data are not available to prove or disprove this within the current review; further work is needed to assess diagnostic performance by deployment site.

### Implications for practice

Ultrasound is clinically practical for confirming PICC line tip position in the NICU. In cases where US is unable to locate malpositioned PICC tips, chest radiography should be performed. Employing US as the reference standard for PICC tip confirmation in the NICU setting would have many advantages. The ability to provide real-time assessment and guidance for repositioning whilst avoiding the use of ionising radiation indicates it is a safe and efficient alternative to conventional radiography. Albeit there are advantages to sonography, particularly in relation to a neonatal population, it is important to acknowledge the potential limitations of US. US is highly operator-dependent [47]. Adoption of bedside US in neonatal practice is gaining popularity; however, widespread implementation is hindered by the lack of unified guidelines and training for US use in neonatal critical care [45].

## Conclusion

Our systematic review and meta-analysis provide evidence that US is sensitive and specific for confirming PICC line placement in the NICU. Our analysis estimated a pooled sensitivity of 95.2% (95% CI: 91.9–97.4%) and a pooled specificity of 71.4% (95% CI: 59.4–81.6%). In cases where US fails to locate misplaced PICC tips, radiography should be performed.
